# Prevalence of Cancer Screening Among Adults With Disabilities, United States, 2013

**DOI:** 10.5888/pcd14.160312

**Published:** 2017-01-26

**Authors:** C. Brooke Steele, Julie S. Townsend, Elizabeth A. Courtney-Long, Monique Young

**Affiliations:** 1 Division of Cancer Prevention and Control, Centers for Disease Control and Prevention, Atlanta, Georgia; 2 Division of Human Development and Disability, Centers for Disease Control and Prevention, Atlanta, Georgia.

## Abstract

**Introduction:**

Many studies on cancer screening among adults with disabilities examined disability status only, which masks subgroup differences. We examined prevalence of receipt of cancer screening tests by disability status and type.

**Methods:**

We used 2013 National Health Interview Survey data to assess prevalence of 1) guideline-concordant mammography, Papanicolaou (Pap) tests, and endoscopy and stool tests; 2) physicians’ recommendations for these tests; and 3) barriers to health-care access among adults with and without disabilities (defined as difficulty with cognition, hearing, vision, or mobility).

**Results:**

Reported Pap test use ranged from 66.1% (95% confidence interval [CI], 60.3%–71.4%) to 80.2% (95% CI, 72.4%–86.2%) among women with different types of disabilities compared with 81.4% (95% CI, 80.0%–82.7%) among women without disabilities. Prevalence of mammography among women with disabilities was also lower (range, 61.2% [95% CI, 50.5%–71.0%] to 67.5% [95% CI, 62.8%–71.9%]) compared with women without disabilities (72.8% [95% CI, 70.7%–74.9%]). Screening for colorectal cancer was 57.0% among persons without disabilities, and ranged from 48.6% (95% CI, 40.3%–57.0%) among those with vision limitations to 64.6% (95% CI, 58.5%–70.2%) among those with hearing limitations. Receiving recommendations for Pap tests and mammography increased all respondents’ likelihood of receiving these tests. The most frequently reported barrier to accessing health care reported by adults with disabilities was difficulty scheduling an appointment.

**Conclusion:**

We observed disparities in receipt of cancer screening among adults with disabilities; however, disparities varied by disability type. Our findings may be used to refine interventions to close gaps in cancer screening among persons with disabilities.

**MEDSCAPE CME**

Medscape, LLC is pleased to provide online continuing medical education (CME) for this journal article, allowing clinicians the opportunity to earn CME credit.

This activity has been planned and implemented through the joint providership of Medscape, LLC and *Preventing Chronic Disease*. Medscape, LLC is accredited by the Accreditation Council for Continuing Medical Education (ACCME), the Accreditation Council for Pharmacy Education (ACPE), and the American Nurses Credentialing Center (ANCC), to provide continuing education for the healthcare team.

Medscape, LLC designates this Journal-based CME activity for a maximum of 1.00 ***AMA PRA Category 1 Credit(s)™***. Physicians should claim only the credit commensurate with the extent of their participation in the activity.

All other clinicians completing this activity will be issued a certificate of participation. To participate in this journal CME activity: (1) review the learning objectives and author disclosures; (2) study the education content; (3) take the post-test with a 75% minimum passing score and complete the evaluation at http://www.medscape.org/journal/pcd; (4) view/print certificate.

**Release date: January 26, 2017; Expiration date: January 26, 2018**

**Learning Objectives**

Upon completion of this activity, participants will be able to:

1. Compare rates of cancer screening among adults with vs those without disability
2. Evaluate how the type of disability might affect rates of cancer screening
3. Analyze the effects of healthcare providers' recommendations for cancer screening
4. Distinguish the most common perceived barrier to healthcare access in the current study

**EDITOR**

Rosemarie Perrin

Editor, *Preventing Chronic Disease*

Disclosure: Rosemarie Perrin has disclosed no relevant financial relationships.CME AUTHOR

**AUTHORS**

C. Brooke Steele, DO

Division of Cancer Prevention and Control, Centers for Disease Control and Prevention, Atlanta, Georgia

Disclosure: C. Brooke Steele, DO, has disclosed no relevant financial relationships.

Julie S. Townsend, MS

Division of Cancer Prevention and Control, Centers for Disease Control and Prevention, Atlanta, Georgia

Disclosure: Julie S. Townsend, MS, has disclosed no relevant financial relationships.

Elizabeth A. Courtney-Long, MA, MSPH

Division of Human Development and Disability, Centers for Disease Control and Prevention, Atlanta, Georgia

Disclosure: Elizabeth A. Courtney-Long, MA, MSPH, has disclosed no relevant financial relationships.

Monique Young, MPH

Division of Cancer Prevention and Control, Centers for Disease Control and Prevention, Atlanta, Georgia

Disclosure: Monique Young, MPH, has disclosed no relevant financial relationships.

**CME AUTHOR**

Charles P. Vega, MD

Health Sciences Clinical Professor of Family Medicine, University of California, Irvine, California

Disclosure: Charles P. Vega, MD, has disclosed the following relevant financial relationships:

Served as an advisor or consultant for: Allergan, Inc.; McNeil Consumer Healthcare

Served as a speaker or a member of a speakers bureau for: Shire

## Introduction

Cancer is the second leading cause of death among adults in the United States (1). Some cancers are preventable with regular screening tests and can be cured if detected and treated early. However, disparities in the use of preventive health services exist. People with disabilities have lower cancer screening rates than people without disabilities (2–7). In 2013, approximately 1 in 5 US adults reported having a disability; disabilities in mobility and cognition were the most frequently reported types (8). People with disabilities may have numerous health-care access barriers, including inaccessible health communication formats, limited access to transportation and parking, and lack of height-adjustable examination tables, accessible mammography equipment, and medical staff trained on proper patient lifting, transferring, and positioning techniques (6,9–11).

People with disabilities are a heterogeneous group whose health needs vary with the types of limitations they have and by the nature of their disabilities (12). Definitions of disability and the level of functioning that qualifies for disability status vary. Many studies on cancer screening among this population used broad disability measures based on limitations in actions within the environment to compare prevalence of screening between people with and without disabilities, and a few disaggregated data by disability severity or type to look at subgroup differences (2–7,9–11,13–16).

In studies of up-to-date breast cancer screening, screening rates ranged from 67% to 79% among women with disabilities and from 70% to 83% among women without disabilities (2,6,9,14). Receipt of up-to-date cervical cancer screening among women with disabilities ranged from 77% to 82%, compared with a range of 83% to 87% among women without disabilities (6,9,14). Breast and cervical cancer screening rates are lower among women with intellectual and developmental disabilities, cognitive disabilities, and multiple disabilities than among women with other disability types (3,5,15). Numerous studies of colorectal cancer (CRC) screening among people with disabilities assessed whether a person was ever screened, rather than whether screening was guideline-concordant (4,5,14,17). Some studies reported that CRC screening rates are higher among people with disabilities than among those without disabilities; however, rates varied by disability type (14,17). Up-to-date CRC screening rates were recently published in a study examining receipt of tests from 1998 through 2010 among people with and without chronic disabilities; the authors reported that receipt was similar between both groups (18). The data in many earlier studies of cancer screening prevalence are now nearly a decade old or older and may not reflect recent trends. Additionally, few studies examined receipt of a health-care provider’s recommendation for screening (11,13,16).

In this study, we used a nationally representative sample to examine differences in receipt of guideline-concordant screening in 2013 for breast cancer, cervical cancer, and CRC by disability status and by type of disability. We report the prevalence of up-to-date breast and cervical cancer screening among women with and without disabilities by screening recommendation status and the prevalence of receiving a recommendation for screening among adults with and without disabilities who were not up-to-date with CRC screening. In addition, we describe perceived barriers to health-care access by disability status.

## Methods

We used data from the 2013 National Health Interview Survey (NHIS), a continuous, cross-sectional survey of US households conducted in-person with noninstitutionalized civilians. NHIS uses trained US Census Bureau interviewers and monitors for quality control. Sampling is done through a complex survey design that involves stratification, clustering, and multiple stages. One sample adult is selected in the household to provide additional information, which in 2013 included information for a cancer control supplement. The final adult response rate for the 2013 survey was 61.2% (19). More information on NHIS design is available at http://www.cdc.gov/nchs/nhis.htm. This study did not require institutional review board approval because we used a publicly available data set without personal identifiers.

We included adults aged 21 to 75 years who provided responses to questions in the NHIS cancer control supplement and the disability module. The disability module consists of 6 questions, the following 4 of which measure serious functional limitations pertaining to hearing, vision, cognition, and mobility (20): Are you deaf or do you have serious difficulty hearing? Are you blind or do you have serious difficulty seeing even when wearing glasses? Because of a physical, mental, or emotional condition, do you have serious difficulty concentrating, remembering, or making a decision? Do you have serious difficulty walking or climbing stairs? In the 2013 survey, these questions were administered to approximately half of sample adults; household members serving as proxies for those who were unable to respond were excluded. Even though survey respondents could report more than one limitation, we excluded those with a mobility disability (eg, difficulty walking or climbing stairs) from the hearing, vision, and cognition categories. We did this because most people with multiple disabilities also have a mobility disability, and we wanted to make our estimates comparable with those in previous disability studies. The hearing, vision, and cognitive categories were not mutually exclusive, and respondents could be in more than one of these categories. Adults who answered no to all disability questions were considered not to have a serious functional limitation. We excluded from the analysis adults who reported having difficulty with self-care (eg, bathing, dressing) or having limitations with community involvement (eg, shopping or running errands alone) but did not report other disabilities (n = 65).

We merged the disability questions file, the sample adult file, and the person file to form the final analytic data set. The merged file had data on 15,079 adults aged 21 to 75 years with known disability status, 12,499 adults without a serious functional disability, and 2,580 adults with at least 1 of the 4 types of disability.

We included the following demographic variables: sex, race/ethnicity, marital status, education level, and general health status. Variables measuring whether respondents had health insurance coverage or usual sources of health care were used to assess health-care access. Barriers to care, such as difficulties with scheduling appointments and reaching clinic staff by telephone, long waiting-room times, inconvenient clinic hours, and lack of transportation served as proxies for barriers to cancer screening. The cancer control supplement included questions about receipt of screening tests for breast cancer, cervical cancer, and CRC. If a survey respondent indicated that he or she had received a screening test, then a follow-up question was asked about the timing of the test. Additionally, women who had seen a clinician within the previous 12 months were asked whether a health-care professional recommended that they receive a mammogram or Papanicolaou (Pap) test. Adults who reported that they were not up to date with screening for CRC were asked whether a health-care provider had recommended that they be tested for problems in their colon or rectum within the previous 12 months. We used US Preventive Services Task Force recommendations that were in place in 2013 to define up-to-date cancer screening. For cervical cancer screening, we assessed whether women aged 21 to 65 years who did not report having had a hysterectomy had received a Pap test within the past 3 years. For breast cancer screening, we assessed whether women aged 50 to 74 years had received a mammogram within the past 2 years. Both men and women aged 50 to 75 years were considered appropriately screened for CRC and current with the screening recommendation if they had received either a fecal occult blood (FOBT) test within the previous year, sigmoidoscopy within the previous 5 years along with FOBT within the previous 3 years, or colonoscopy within the previous 10 years.

We used SAS 9.3 (SAS Institute Inc) and SAS-callable SUDAAN release 11 (Research Triangle Institute) to conduct all analyses. All prevalence estimates were weighted so that they represent the noninstitutionalized, civilian US population. The final survey weights account for race/ethnicity, sex, and age composition of that population.

We calculated the weighted prevalence estimates with 95% confidence intervals (CIs) for demographic characteristics, health-care access, and cancer screening (including by provider recommendation) among adults without a disability; separately for adults with hearing, vision, cognition, or mobility limitations; and for all adults with any of these disability types. We conducted multivariable logistic regression analysis to obtain adjusted odds ratios for the likelihood of being up-to-date for cancer screening for each type of disability (adults without disability served as the referent group for all models), while controlling for the influence of demographic characteristics and health-care access. In all models, we controlled for race/ethnicity, marital status, education level, health-care coverage, general health status (excluded in the hearing disability model for CRC screening because of statistical nonsignificance), and usual source of care. The cervical cancer screening model also controlled for age category, and the CRC screening model controlled for age category and sex. We used a backward elimination approach to select variables for the final models and assessed goodness of fit using the Hosmer–Lemeshow test. Variables with *P* ≥ .10 from the Wald *F* test were eliminated unless they traditionally appeared in cancer screening models.

## Results

Overall, 16.9% of adults aged 21 to 75 years were identified as having at least 1 of the 4 disability types. The prevalence of each disability type among this sample was mobility, 57.9%; cognition, 18.2%; hearing, 18.8%; and vision, 11.2%. Thirty-two percent of persons had more than one disability, with most (82.8%) having mobility limitations in combination with another disability. Regardless of disability type or status, most adults were non-Hispanic white and had health insurance coverage and a usual source of care (Table 1). Compared with persons with no disability (6.4%), the prevalence of reporting fair or poor health was substantially higher among persons with a mobility disability (63.3%), a cognitive disability (38.4%), a vision disability (29.4%), or a hearing disability (17.4%).

**Table 1:** Prevalence of Selected Demographic Characteristics and Health Behaviors Among Adults Aged 21–75 Years (N = 15,079), by Disability Type^a^, National Health Interview Survey, United States, 2013

Characteristic	Type of Disability, % (95% Confidence Interval)					
	Hearing	Vision	Cognitive	Mobility	Any Type	None
**Total**	454	304	471	1,512	2,580	12,499
**Sex**						
Male	62.5 (57.2–67.6)	47.9 (41.5–54.3)	47.8 (42.2–53.4)	43.4 (40.4–46.4)	47.5 (45.3–49.7)	46.8 (45.7–47.9)
Female	37.5 (32.4–42.8)	52.1 (45.7–58.5)	52.2 (46.6–57.8)	56.6 (53.6–59.6)	52.5 (50.3–54.7)	53.2 (52.1–54.3)
**Age group, y**						
21–49	27.3 (23.0–32.1)	42.7 (36.2–49.5)	53.6 (48.2–59.0)	21.3 (18.9–24.0)	29.7 (27.6–31.9)	60.9 (59.5–61.7)
50–64	35.7 (31.1–40.6)	36.1 (30.2–42.4)	33.0 (28.0–38.4)	46.5 (43.7–49.4)	41.4 (39.3–43.7)	27.0 (26.0–27.9)
65–75	37.0 (32.2–42.0)	21.2 (16.2–27.3)	13.4 (10.4–17.0)	32.2 (29.4–35.1)	28.9 (26.9–30.9)	12.4 (11.7–13.2)
**Race/ethnicity**						
Non-Hispanic white	81.2 (77.3–84.5)	60.5 (53.8–66.7)	70.6 (65.9–74.9)	66.1 (63.1–68.9)	69.4 (67.2–71.5)	66.7 (65.6–67.7)
Non-Hispanic black	5.8 (4.1–8.2)	14.7 (10.8–19.6)	14.8 (12.0–18.1)	18.4 (16.1–21.0)	15.1 (13.5–16.9)	11.9 (11.2–12.6)
Hispanic	7.7 (5.6–10.6)	18.6 (14.4–23.7)	9.9 (7.4–13.2)	10.7 (9.1–12.5)	10.6 (9.3–12.1)	14.0 (13.3–14.7)
Other^b^	5.3 (3.6–7.7)	—c	4.8 (3.2–7.1)	4.9 (3.8–6.2)	4.9 (4.0–5.9)	7.4 (6.9–8.0)
**Marital status**						
Married/living together	51.0 (45.4–56.5)	37.9 (32.1–44.0)	29.0 (24.3–34.3)	40.1 (37.0–43.2)	40.5 (38.2–42.9)	54.8 (53.6–56.0)
Single/never married	17.5 (13.6–22.2)	22.1 (16.9–28.4)	35.9 (30.7–41.4)	16.4 (14.3–18.7)	19.9 (18.0–21.9)	24.6 (23.5–25.8)
Divorced/widowed/ separated	31.5 (26.5–37.0)	40.0 (34.1–46.2)	35.1 (30.2–40.2)	43.5 (40.6–46.5)	39.6 (37.4–41.8)	20.5 (19.7–21.4)
**Education**						
Less than high school diploma	18.4 (14.5–23.0)	22.7 (18.0–28.3)	22.4 (18.9–26.4)	25.1 (22.4–28.0)	23.2 (21.4–25.1)	9.9 (9.3–10.5)
High school diploma/GED	24.9 (20.6–29.8)	32.5 (27.0–38.5)	31.3 (27.0–36.0)	31.0 (28.0–34.3)	30.0 (27.8–32.3)	24.1 (23.1–25.1)
Some college/ associates degree	30.9 (26.1–36.2)	26.3 (21.2–32.1)	29.5 (24.9–34.5)	31.1 (28.2–34.1)	30.1 (28.1–32.2)	31.2 (30.2–32.3)
Bachelor’s degree or higher	25.8 (21.1–31.3)	18.5 (13.6–24.6)	16.8 (12.9–21.5)	12.8 (10.8–15.1)	16.7 (15.0–18.5)	34.8 (33.6–36.0)
**General health status**						
Excellent/very good	47.5 (42.0–53.2)	37.3 (30.6–44.5)	23.5 (19.1–28.7)	11.7 (9.8–13.8)	22.8 (20.7–25.0)	68.6 (67.6–69.5)
Good	35.1 (30.2–40.4)	33.4 (27.8–39.5)	38.1 (33.3–43.0)	25.1 (22.6–27.7)	29.4 (27.5–31.5)	25.1 (24.2–26.0)
Fair/poor	17.4 (13.4–22.2)	29.4 (23.9–35.5)	38.4 (33.3–43.8)	63.3 (60.4–66.1)	47.8 (45.3–50.2)	6.4 (5.9–6.9)
**Has insurance coverage**						
Yes	86.5 (82.7–89.6)	80.7 (75.7–84.9)	81.9 (77.1–85.9)	89.4 (87.4–91.1)	87.0 (85.4–88.4)	82.3 (81.5–83.1)
**Has usual source of care**						
Yes	85.9 (81.8–89.2)	79.5 (72.6–85.1)	88.3 (84.4–91.3)	93.8 (92.2–95.1)	90.3 (88.8–91.6)	82.8 (81.9–83.6)

Abbreviation: GED, general educational development.

a Respondents could report more than one limitation and were included in the analysis for each reported limitation, with the exception of mobility limitations. Regardless of any additional limitation, people with mobility limitations were only included in the mobility limitation subgroup.

b American Indian/Alaska Native, Asian, multiple races, and race group not releasable.

c Estimate suppressed because relative standard error was greater than 30%.

In unadjusted prevalence estimates (Table 2), women with any of the disability types had a lower prevalence of reporting up-to-date cervical and breast cancer screening than women without a disability. However, men and women with any of the disability types had a slightly higher prevalence of up-to-date CRC screening than adults without disabilities. Women with mobility limitations had the lowest prevalence (66.1%) of receiving a Pap test within the past 3 years, whereas those with cognitive limitations had the highest (80.2%). The reverse was true for receipt of mammograms within the past 2 years; prevalence was lower among women with cognitive limitations (61.2%) and higher among those with mobility limitations (67.5%). When controlling for sociodemographic and health-care–related variables we found that compared with women without disability, the odds of receiving a Pap test within the previous 3 years were significantly lower among women with any of the disability types (AOR, 0.77; 95% CI, 0.60–0.99) and among women with a mobility limitation (AOR, 0.58; 95% CI, 0.42–0.80). The odds of receiving mammograms within the previous 2 years were also lower among women with any of the disability types; however, this finding was not significant.

**Table 2:** Prevalence and Adjusted Odds Ratios^a^ for Up-to-Date Cancer Screening Among Adults With a Disability Compared With Adults With No Disability, National Health Interview Survey, United States, 2013

Characteristic	Disability Type^b^					
	Hearing	Vision	Cognitive	Mobility	Any Type	None
**Pap test**						
Pap test within past 3 years^c^, n	90	94	166	392	700	5,184
Yes	73.1 (61.9–81.9)	76.6 (64.8–85.3)	80.2 (72.4–86.2)	66.1 (60.3–71.4)	71.5 (67.4–75.2)	81.4 (80.0–82.7)
AOR for up-to-date status	0.82 (0.44–1.53)	0.99 (0.51–1.90)	1.20 (0.71–2.03)	0.58 (0.42–0.80)	0.77 (0.60–0.99)	Reference
**Mammogram**						
Mammogram within past 2 years^d^, n	115	80	107	633	897	2,544
Yes	66.5 (55.4–76.1)	63.7 (50.4–75.2)	61.2 (50.5–71.0)	67.5 (62.8–71.9)	66.7 (63.0–70.2)	72.8 (70.7–74.9)
AOR for up-to-date status	0.89 (0.53–1.49)	0.88 (0.48–1.61)	0.91 (0.54–1.55)	1.04 (0.77–1.40)	0.97 (0.76–1.25)	Reference
**Colorectal cancer screening**						
Colorectal cancer screening^e^>, n	320	175	216	1,136	1,746	4,726
Yes	64.6 (58.5–70.2)	48.6 (40.3–57.0)	56.2 (47.9–64.2)	63.1 (60.0–66.1)	61.8 (59.1–64.5)	57.0 (55.3–58.6)
Received colonoscopy^f^, n	93.1 (87.5–96.3)	92.8 (84.5–96.8)	93.6 (86.3–97.2)	93.5 (91.2–95.3)	93.5 (91.5–95.0)	94.7 (93.7–95.6)
AOR for up-to-date status	1.41 (1.04–1.91)	0.90 (0.60–1.35)	1.25 (0.84–1.85)	1.33 (1.12–1.58)	1.29 (1.10–1.52)	Reference

Abbreviation: AOR, adjusted odds ratio; Pap, Papanicolaou.

a Adjusted odds ratios are from logistic regression analyses that examined disability status in relation to being up-to-date on cancer screening tests while controlling for race/ethnicity, insurance, having a usual source of health care, general health status, marital status, and education. Colorectal cancer screening models also included sex and age category. General health status was not included in the hearing disability model because it was not significant. Pap test models also were controlled for age category.

b Values are percentage (95% confidence interval) unless otherwise stated.

c Women aged 21 to 65 years who had a Pap test within the past 3 years. Data on Pap tests were available for 5,884 women.

d Women aged 50 to 74 years who had a mammogram within the past 2 years. Data on mammograms were available for 3,441 women.

e Adults aged 50 to 75 years who had a high-sensitivity fecal occult blood test within the past 12 months, a sigmoidoscopy within the past 5 years with a high-sensitivity fecal occult blood test within the past 3 years, or a screening colonoscopy within the past 10 years. Data on colorectal cancer screening were available for 6,472 men and women.

f Colonoscopy within the past 10 years among adults with known disability status who reported being up-to-date with colorectal cancer screening (n = 3,677).

The prevalence of up-to-date breast and cervical cancer screening was higher among women who reported receiving recommendations from their health-care providers for these tests than among women who said they had not received recommendations (Figure). Among women who reported receiving recommendations, those with any of the disability types were less likely than those without any of the disability types to report receiving Pap tests within the previous 3 years (84.1% vs 94.7%, respectively, *P* < .001). Among women who indicated that they had not received recommendations, those with any of the disability types reported less use of mammography (32.6% vs 48.6%, *P* < .001) and of Pap tests (55.2% vs 66.7%, *P* = .003) than women without any of the disability types. Among adults who reported that they were not up-to-date with screening for CRC, the percentage who had received recommendations for CRC screening tests in the previous 12 months was 15.2% for those with any of the disability types and 11.9% for those without any of the disability types (*P* > .05) (data not shown). The prevalence of perceived barriers to accessing health care was higher among persons with any of the disability types than persons without any of the disability types; the most frequently cited barrier among both groups was difficulty getting a clinic appointment (9.1% vs 4.5%) (Table 3).

**Figure:**
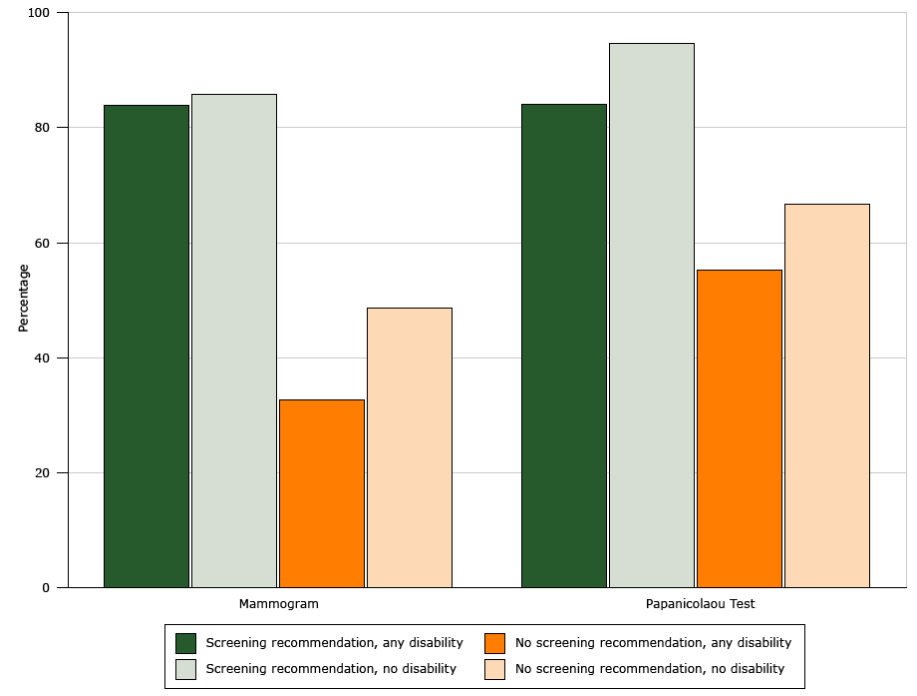
Prevalence of up-to-date cancer screening among women, by disability status and whether or not a doctor or health professional recommended the screening test, National Health Interview Survey, United States, 2013. **Table:** 

Screening	Mammogram	Pap test
Screening recommendation for those with any disability (hearing, vision, cognitive, or mobility)	83.9	84.1
Screening recommendation for those with no disability	85.8	94.7
No screening recommendation for those with any disability (hearing, vision, cognitive, or mobility)	32.6	55.2
No screening recommendation for those with no disability	48.6	66.7

**Table 3:** Perceived Barriers to Health Care Access by Disability Status (N = 12,499^a^), National Health Interview Survey, United States, 2013

Barrier	Any Disability^b^	No Disability
	% (95% Confidence Interval)	
Difficulty getting through on telephone to reach clinic	4.2 (3.4–5.2)	1.6 (1.4–1.9)
Difficulty getting clinic appointment	9.1 (7.9–10.6)	4.5 (4.1–4.9)
Wait time at clinic too long	7.3 (6.1–8.7)	2.8 (2.5–3.2)
Clinic not open at convenient times	4.3 (3.5–5.4)	2.3 (2.0–2.6)
No available transportation to clinic	6.5 (5.4–7.8)	0.8 (0.7–1.0)

a The denominator for each survey question varies because of the exclusion of persons with unknown and missing responses.

b Includes hearing, vision, cognitive, or mobility disability.

## Discussion

Overall, our findings on cancer screening among persons with disabilities are consistent with some previous research on this topic (2,3,14,16). We found a lower prevalence of breast and cervical cancer screening among women with disabilities, but only the disparity in Pap test use persisted in models adjusted for cancer screening-related variables. A similar pattern was reported in a study on use of mammography and Pap tests by disability status and severity (3). In a study examining receipt of these tests among women with and without limitations in activities of daily living, however, disparities remained significant in multivariable analyses for each test (14). Similar to previous research on CRC screening, we found slightly higher rates of screening among adults with disabilities than among those without disabilities (14,18). Even though persons with disabilities were reported to have higher CRC screening rates than persons without disabilities from 1998 to 2010, the rates reported in 2010 (59.2% vs 58.9%) and in our study (61.8% vs 57%) are below the 70.5% *Healthy People 2020* objective (https://www.healthypeople.gov/2020/topics-objectives/topic/cancer/objectives) (18). The breast and cervical cancer screening rates in our study are also lower than national objectives for these tests (81.1% for breast cancer, 93.0% for cervical cancer). Future studies should explore the reasons why some persons with disabilities may avoid or delay cancer screening, including the extent to which factors such as disability type, severity of limitation, and confusion about screening guidelines affect decision making. Research on this topic is scarce, especially for cervical cancer and CRC.

We found that the prevalence of breast and cervical cancer screening was higher among women who reported receiving recommendations for these tests from their health-care providers, which is consistent with previous studies (11,13). Women with disabilities were slightly less likely than those without disabilities to report receipt of screening recommendations, but the differences were not significant. Less than 15% of persons who were not up-to-date with CRC screening reported receiving recommendations for these tests, regardless of disability status. We did not find other studies that examined receipt of recommendations for CRC screening among this population. Additional research is needed to help identify the reasons why health-care providers might not refer persons with disabilities for cancer screening. Some providers may prioritize managing the disabling condition and related illnesses to the exclusion of addressing preventive health needs (10,14). Tools have been developed to help clinicians identify recommended preventive services and increase their use among persons with disabilities (21–23). Information is also available for health insurers, community-based organizations, and health educators to help increase their knowledge about disability and health and identify service gaps (21).

We found that most people with disabilities had health insurance coverage and a usual source of health care. This finding is consistent with previous research and may be related to links between enrollment in federal and state disability benefits programs and eligibility for public health insurance (2,3,8,24–26). However, some insured people with disabilities reported unmet health-care needs and difficulty accessing care. These disparities may be related to disability type (eg, mobility limitations), or they may be unrelated to disability status (eg, out-of-pocket cost) (24,27). In our study, people with disabilities were 7 times as likely to report transportation barriers as those without disabilities and nearly twice as likely to report difficulty scheduling appointments, long wait times at clinics, and inconvenient clinic hours.

Our study has limitations. NHIS data are based on self-report and may be subject to recall bias, particularly among persons with serious cognitive disabilities, and subject to social desirability bias. The questions used to measure disability in the NHIS disability module, however, have been cognitively tested by the National Center for Health Statistics and the US Census Bureau (28). Additionally, self-reported data for mammography, Pap tests, and CRC screening are reported to be reliable (29,30). The small sample sizes for some disability subgroups limit the generalizability of our findings, but the sizes all met criteria for analysis; the data are also weighted to be representative of the US population. Nearly one-third of respondents had more than one type of disability; therefore, our results might not be generalizable to those with a single disability. Because we could not distinguish between tests received for diagnostic purposes and tests received for screening purposes, actual cancer screening rates may be lower than reported. We also could not assess the onset of disability in relation to receipt of screening tests. Using data based on disability status at the time of data collection rather than the time of screening could have resulted in overestimates of screening among those whose disabilities were diagnosed after they were screened. Furthermore, because the NHIS disability survey includes only noninstitutionalized adults living in the community, we could not assess the prevalence of cancer screening among adults living in group homes and other settings in which the prevalence of disability may be higher.

One of the strengths of our study is that we used population-based data. We also examined 2 cancer screening-related topics that have been rarely researched among persons with disabilities — reported receipt of guideline-concordant CRC screening and recommendations by health-care providers for cancer screening.

Disparities among persons with disabilities in receipt of preventive services and health-care access persist. Our study findings may be used to increase awareness about gaps in cancer screening among subgroups of this population, to inform development of interventions that educate people with disabilities about the importance of discussing preventive health services with their health care providers, and to remind providers about the critical roles they play in recommending use of these services.

## Post-Test Information

To obtain credit, you should first read the journal article. After reading the article, you should be able to answer the following, related, multiple-choice questions. To complete the questions (with a minimum 75% passing score) and earn continuing medical education (CME) credit, please go to http://www.medscape.org/journal/pcd. Credit cannot be obtained for tests completed on paper, although you may use the worksheet below to keep a record of your answers. You must be a registered user on Medscape.org. If you are not registered on Medscape.org, please click on the "Register" link on the right hand side of the website to register. Only one answer is correct for each question. Once you successfully answer all post-test questions you will be able to view and/or print your certificate. For questions regarding the content of this activity, contact the accredited provider, CME@medscape.net. For technical assistance, contact CME@webmd.net. American Medical Association’s Physician’s Recognition Award (AMA PRA) credits are accepted in the US as evidence of participation in CME activities. For further information on this award, please refer to http://www.ama-assn.org/ama/pub/about-ama/awards/ama-physicians-recognition-award.page. The AMA has determined that physicians not licensed in the US who participate in this CME activity are eligible for ***AMA PRA Category 1 Credits™***. Through agreements that the AMA has made with agencies in some countries, AMA PRA credit may be acceptable as evidence of participation in CME activities. If you are not licensed in the US, please complete the questions online, print the AMA PRA CME credit certificate and present it to your national medical association for review.

## Post-Test Questions

### Study Title: Prevalence of Cancer Screening Among Adults With Disabilities, United States, 2013

#### CME Questions

1. You are seeing a 53-year-old woman with a history of cerebral palsy and intellectual disability. She has chronic mild to moderate disabilities related to these conditions. According to the current study by Steele and colleagues, screening for which of the following cancer types was ***more*** likely to be up-to-date among adults with disabilities vs those without disabilities?
  - Colorectal cancer
  - Cervical cancer
  - Breast cancer
  - Lung cancer
2. A review of the patient's record indeed indicates that she is behind on screening requirements for breast and cervical cancer. How did the type of disability affect screening rates for these cancers in the current study?
  - Mobility limitations were associated with the lowest rates of cervical cancer screening; cognitive limitations were associated with the lowest rates of breast cancer screening
  - Cognitive limitations were associated with the lowest rates of both cervical and breast cancer screening
  - Mobility limitations were associated with the lowest rates of both cervical and breast cancer screening
  - Cognitive limitations were associated with the lowest rates of cervical cancer screening; mobility limitations were associated with the lowest rates of breast cancer screening
3. You recommend that she receive breast and cervical cancer screening. According to the current study, which of the following statements regarding healthcare recommendations for cancer screening for adults with disabilities is ***most*** accurate?
  - Recommendations were not associated with higher rates of breast cancer screening
  - Recommendations were not associated with higher rates of cervical cancer screening
  - Recommendations were more effective for promoting cervical cancer screening among women with a disability compared with women without disabilities
  - Among women who did not receive a recommendation, women with disabilities had lower rates of mammography and Pap tests compared with women without disabilities
4. The patient is hesitant to follow through on your recommendations. What was the ***most*** commonly cited barrier to healthcare access in the current study?
  - Too long of a wait at the clinic
  - Clinic not open at convenient times
  - No available transportation to clinic
  - Difficulty getting a clinic appointment

**Table:** Evaluation

**1. The activity supported the learning objectives.**				
**Strongly Disagree**				**Strongly Agree**
**1**	**2**	**3**	**4**	**5**
**2. The material was organized clearly for learning to occur.**				
**Strongly Disagree**				**Strongly Agree**
**1**	**2**	**3**	**4**	**5**
**3. The content learned from this activity will impact my practice.**				
**Strongly Disagree**	** **	** **	** **	**Strongly Agree**
**1**	**2**	**3**	**4**	**5**
**4. The activity was presented objectively and free of commercial bias.**				
**Strongly Disagree**				**Strongly Agree**
**1**	**2**	**3**	**4**	**5**
